# Hippocampal Neuroinflammation, Functional Connectivity, and Depressive Symptoms in Multiple Sclerosis

**DOI:** 10.1016/j.biopsych.2015.11.022

**Published:** 2016-07-01

**Authors:** Alessandro Colasanti, Qi Guo, Paolo Giannetti, Matthew B. Wall, Rexford D. Newbould, Courtney Bishop, Mayca Onega, Richard Nicholas, Olga Ciccarelli, Paolo A. Muraro, Omar Malik, David R. Owen, Allan H. Young, Roger N. Gunn, Paola Piccini, Paul M. Matthews, Eugenii A. Rabiner

**Affiliations:** a Division of Brain Sciences, Department of Medicine, Imperial College London, London, United Kingdom; b Centre for Affective Disorders, Institute of Psychiatry, Psychology and Neuroscience, King’s College London, London, United Kingdom; c Psychological Medicine, and Centre for Neuroimaging Sciences, Institute of Psychiatry, Psychology and Neuroscience, King’s College London, London, United Kingdom; d Imanova Centre for Imaging Sciences, London, United Kingdom; e Imperial College Healthcare National Health Service Trust, London, United Kingdom; f Department of Neuroinflammation, University College London Institute of Neurology, London, United Kingdom; g National Institute of Health Research Biomedical Research Centre at University College London Hospitals, London, United Kingdom

**Keywords:** Depression, Hippocampus, Multiple sclerosis, Neuroimaging, Neuroinflammation, TSPO

## Abstract

**Background:**

Depression, a condition commonly comorbid with multiple sclerosis (MS), is associated more generally with elevated inflammatory markers and hippocampal pathology. We hypothesized that neuroinflammation in the hippocampus is responsible for depression associated with MS. We characterized the relationship between depressive symptoms and hippocampal microglial activation in patients with MS using the 18-kDa translocator protein radioligand [^18^F]PBR111. To evaluate pathophysiologic mechanisms, we explored the relationships between hippocampal neuroinflammation, depressive symptoms, and hippocampal functional connectivities defined by resting-state functional magnetic resonance imaging.

**Methods:**

The Beck Depression Inventory (BDI) was administered to 11 patients with MS and 22 healthy control subjects before scanning with positron emission tomography and functional magnetic resonance imaging. We tested for higher [^18^F]PBR111 uptake in the hippocampus of patients with MS relative to healthy control subjects and examined the correlations between [^18^F]PBR111 uptake, BDI scores, and hippocampal functional connectivities in the patients with MS.

**Results:**

Patients with MS had an increased hippocampal [^18^F]PBR111 distribution volume ratio relative to healthy control subjects (*p* = .024), and the hippocampal distribution volume ratio was strongly correlated with the BDI score in patients with MS (*r* = .86, *p* = .006). Hippocampal functional connectivities to the subgenual cingulate and prefrontal and parietal regions correlated with BDI scores and [^18^F]PBR111 distribution volume ratio.

**Conclusions:**

Our results provide evidence that hippocampal microglial activation in MS impairs the brain functional connectivities in regions contributing to maintenance of a normal affective state. Our results suggest a rationale for the responsiveness of depression in some patients with MS to effective control of brain neuroinflammation. Our findings also lend support to further investigation of the role of inflammatory processes in the pathogenesis of depression more generally.

There is a higher prevalence of depression in patients with multiple sclerosis (MS) than in the general population (1). The association between MS and depression is stronger than associations observed in patients with other long-term disabling conditions, suggesting common pathophysiologic mechanisms (2). Activation of the brain innate immune response has been proposed as one such potential common causal factor (3).

Magnetic resonance imaging (MRI) studies have reported associations between depressive symptoms in patients with MS and measures of disease burden, including lesion load and accompanying tissue destruction, more diffuse abnormalities of the normal-appearing white matter, and brain atrophy (4). However, each of these associations accounted for only a small amount of total variance in depressive symptoms, and specific associations have not been consistent across studies. These findings suggest that the association arises from a common underlying factor that contributes to pathophysiologic changes for both MS and depression.

Elevated inflammatory markers are associated with depressive symptoms in medically healthy individuals (5), and increased levels of depression and anxiety have been documented in clinical and experimental settings after challenges that activate an innate immune response (6, 7). We hypothesized that the high prevalence of depressive symptoms in patients with MS is a direct consequence of chronic innate immune activation in functionally relevant regions of their brains.

Convergent lines of research implicate the hippocampus in the pathophysiology of depression. Hippocampal atrophy is a consistently reported finding in patients with depression and has been suggested as a biomarker of risk for depression (8). Hippocampal activity modulates the hypothalamus-pituitary-adrenal stress hormone axis and regulates the function of prefrontal, ventral tegmental, and striatal areas that appear to contribute to the genesis or maintenance of depressive symptoms (9). The hippocampus may be particularly susceptible to neuroinflammatory triggers; for example, it has a high density of interleukin-1 receptors (10). Hippocampal pathology in MS, including extensive demyelination, neuronal loss atrophy, and microglia activation, has been confirmed by postmortem and imaging studies (11, 12, 13). Studies using MRI demonstrated that hippocampal volume loss and altered morphology are associated with depressive symptoms in MS (14, 15, 16). Hippocampal neuroinflammation in the rodent experimental allergic encephalomyelitis (EAE) model is associated with dysfunctional neurogenesis (17), and hippocampal neurogenesis in humans may be critical to recovery from depression (18). Therefore, we hypothesized that innate immune responses specifically in the hippocampus are responsible for the genesis of depressive symptoms associated with MS.

We previously demonstrated that positron emission tomography (PET) with the second-generation 18-kDa translocator protein (TSPO) radioligand [^18^F]PBR111 enables the characterization of microglial activation in the white matter of patients with MS (19). Although TSPO is not seen exclusively in activated microglia, we interpret increased TSPO signal as arising largely from activated microglia/macrophages based on previous immunohistochemical observations in postmortem brains with MS and in EAE rodents (20, 21, 22). Studies in patients with MS using first-generation and second-generation TSPO radioligands reported focally increased TSPO radioligand uptake associated with gadolinium-enhancing lesions (23, 24), some T2-hyperintense lesions (19, 25), in the thalamus (23, 25), and in some cortical gray matter areas, particularly in patients with secondary progressive MS (26).

In the present study, we used [^18^F]PBR111 PET to quantify hippocampal microglia activation and characterize its relationship to depressive symptoms in patients with MS in vivo. We also investigated whether the strength of hippocampal functional connectivity assessed with resting-state functional MRI is related to the expression of depressive symptoms and to hippocampal microglia activation in patients with MS.

## Methods and Materials

### Study Design and Subjects

The study was conducted at Imperial College London and the Imanova Centre for Imaging Sciences and was approved by the Essex 1 Research Ethics Committee and the Administration of Radioactive Substances Advisory Committee. The clinical, demographic, and radiologic characteristics of study subjects are summarized in Supplemental Table S1 and Table 1. The study subjects included 22 healthy control subjects and 13 patients with relapsing-remitting MS who underwent MRI and [^18^F]PBR111 PET scans on the same day. The MRI scan was conducted approximately 2 hours before the PET scan. All healthy control subjects underwent a PET scan, and 14 of 22 also underwent a resting-state functional MRI scan. Of 13 patients with MS, 2 were subsequently excluded from the PET cohort because of failure of metabolite analysis, which precluded the kinetic modeling using arterial input function with metabolite correction.

The mean age of patients with MS was younger than healthy control subjects (MS group, 44.23 ± 8.42 years [mean ± SD]; healthy control group, 49.77 ± 11.07 years; *t* = 1.55, *p* = .13), and there were more women in the MS group than in the healthy control group (MS group, 11/13 female [84.6%]; healthy control group, 14/22 female [63.6%]; χ^*2*^ = 1.76, *p* = .18). Of 13 patients with MS, 6 were taking antidepressant medications and 10 were receiving disease-modifying treatments at the time of the examinations (Supplemental Table S1). Subjects were stratified into one of three binding affinity groups (high-affinity binders, mixed-affinity binders, low-affinity binders) on the basis of their genotype for the rs6971 polymorphism, which is a major determinant of variations in affinity for second-generation TSPO radioligands between subjects (27).

### Clinical Assessments

Clinical assessments were performed at screening. Disability was assessed using the Expanded Disability Status Scale (EDSS) (28). Diagnoses of major depressive episode (MDE) were formulated by an experienced psychiatrist on the basis of the Mini International Neuropsychiatric Interview (29). Current and recent (within the past 6 months) MDEs were recorded. The Beck Depression Inventory (BDI)-II (30) was used for assessment of depressive symptoms, and fatigue was assessed using the Fatigue Severity Scale (31).

### PET Imaging

The PET radioligand synthesis, image acquisition protocol, definition of regions of interest (ROIs), and quantification of [^18^F]PBR111 are described in detail in Appendix A, Appendix A. The hippocampus was chosen a priori as the primary ROI for [^18^F]PBR111 binding analyses. The thalamus was used as a control ROI, based on its potential for accurate segmentation and high TSPO signal (32), to test whether any observed increase in TSPO was specific to hippocampus or global. A post hoc exploratory analysis further tested for group differences in all major ROIs (details in Appendix A, Appendix A).

The [^18^F]PBR111 total volume of distribution (V_T_) was quantified using a two-tissue compartment model with metabolite-corrected arterial input function (32). The relative regional [^18^F]PBR111 binding (as an index of activated microglia density) was estimated by calculating the distribution volume ratio (DVR), which was defined as the ratio of [^18^F]PBR111 V_T_ in a ROI to the [^18^F]PBR111 V_T_ across the entire cortical gray matter used as a “pseudo-reference region.” The use of the DVR reduces variability associated with nonspecific binding of the radiotracer by minimizing errors associated with the estimation of the blood input function. Normalization is associated with an improved test-retest reproducibility of [^18^F]PBR111 signal (19), leading to reduced within-subject variability and to a better signal-to-noise ratio.

### Resting-State Functional MRI

The MRI data were acquired on a 3-tesla Siemens Verio (Siemens Healthcare, Erlangen Germany) clinical MRI scanner, equipped with a 32-channel phased-array head coil. The MRI protocols included T1-weighted (with and without gadolinium), T2-weighted fluid attenuated inversion recovery, and resting-state functional MRI. Details on the MRI protocol and functional connectivity analysis are presented in Appendix A, Appendix A.

### Statistical Analyses

Statistical analyses were done using IBM SPSS Statistics version 20 (IBM Corp., Armonk, New York). The analyses were divided into group analysis, exploratory association analysis, and functional connectivity analysis. A detailed description of statistical analyses is provided in the Appendix A, Appendix A.

Group analyses were performed using *t* tests for independent samples and analysis of covariance with DVR or V_T_ as dependent variables, group (or diagnosis of MDE) and rs6971 genotype as fixed factors, and age as covariate. Exploratory association analyses were performed using Pearson partial correlation, while controlling for age, EDSS scores, and disease duration. The BDI items were classified into two clusters, cognitive and somatic, based on the symptom components most commonly identified in studies on the factor structure of BDI-II (33).

For the functional connectivity analysis, a bilateral hippocampal ROI was used as the seed region. Hippocampal time-series data were extracted from the functional data, and contrasts modeled the positive and negative effects of the hippocampal time-series regressor. For the analysis of correlations of hippocampal functional connectivity in patients with MS, hippocampal [^18^F]PBR111 DVR and BDI were the regressors of interest in the group-level model, whereas nuisance regressors included age, disease duration, and EDSS. All statistical images from the group models were thresholded at *p* < .05 (or *z* = 2.3; cluster-corrected for multiple comparisons).

## Results

### Group Analysis

Patients with MS had a higher mean BDI score than healthy control subjects (MS group, 15 ± 8.63 [mean ± SD]; healthy control group, 1.41 ± 2.75; *t* = 6.9, *p* < .0001). Of 11 patients with MS, 6 (54%) met criteria for current diagnosis of MDE; in 3 cases (27%), patients met criteria for a recent MDE (within last 6 months) but did not have active symptoms at the time of examination. None of the healthy control subjects had a current or past MDE. The BDI was correlated with the EDSS (Pearson *r* = .72; *p* = .013) for patients with MS. There was no correlation between BDI and age or between BDI and duration of disease.

**Figure 1:**
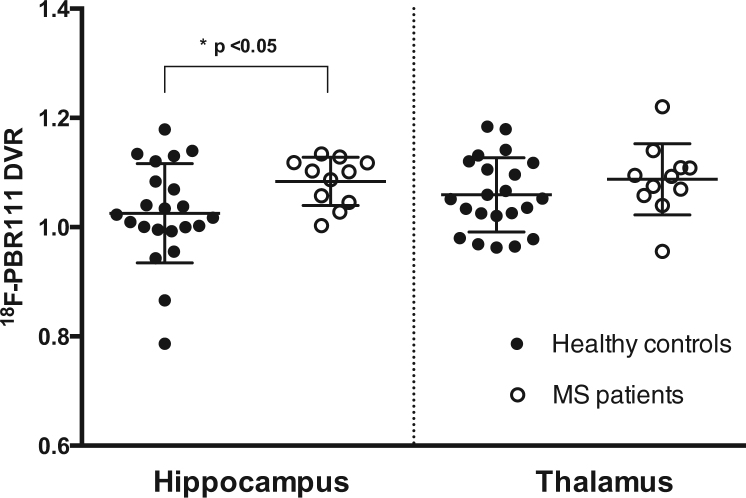
Comparison of [^18^F]PBR111 distribution volume ratio (DVR) between groups in the regions of interest. The [^18^F]PBR111 DVR was higher in patients with MS (multiple sclerosis) than healthy control subjects in the hippocampus, but not in the thalamus. Each circle represents individual subjects’ DVR values. The horizontal lines represent the group’s mean and SD.

A whole-brain analysis of the distribution [^18^F]PBR111 signal was reported previously (32). The [^18^F]PBR111 DVR was higher in the hippocampus of patients with MS relative to healthy control subjects (MS group, 1.083 ± 0.04 [mean ± SD]; healthy control group, 1.025 ± 0.09; *t* = 2.49, *p* = .018) (Figure 1). The difference remained significant (*F* = 5.73, *p* = .024) after correction for age and rs6971 genotype. The rs6971 genotype did not affect differences between groups and was not associated with hippocampal [^18^F]PBR111 DVR. Age correlated with hippocampal [^18^F]PBR111 DVR (*F* = 4.39, *p* = .046). No significant difference between patients with MS and healthy control subjects was found in the thalamus (*F* = .66, *p* = .42) (Figure 1). The [^18^F]PBR111 V_T_ in the cortical gray matter (which was used as a pseudo-reference region for DVR contrasts) also was similar between patients with MS and healthy control subjects, even after correction for age and rs6971 genotype (*F* = .01, *p* = .98).

### Exploratory Association Analyses

**Figure 2:**
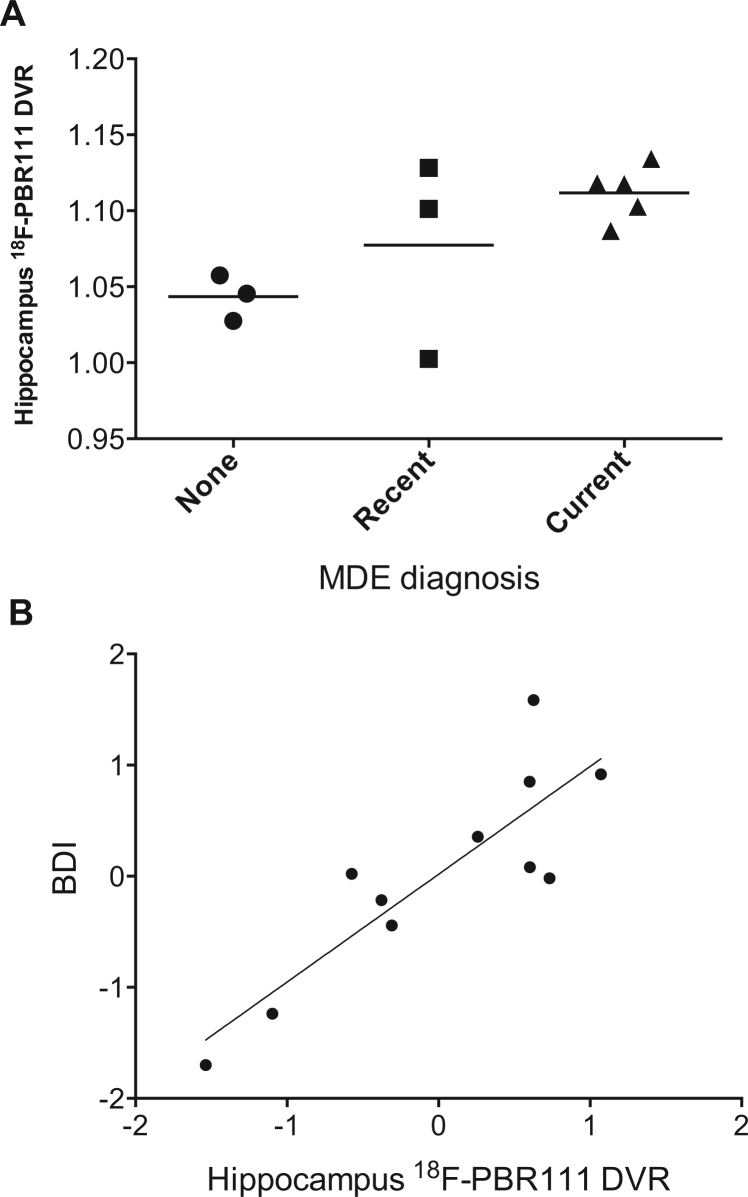
Relationship between hippocampal [^18^F]PBR111 distribution volume ratio (DVR) and depression (major depressive episode [MDE] diagnosis and Beck Depression Inventory [BDI] scores) in patients with multiple sclerosis. **(A)** Relationship between diagnosis of MDE and [^18^F]PBR111 DVR in the hippocampus. Recent MDE diagnosis refers to the occurrence of a MDE in the last 6 months that has resolved at the time of scanning. **(B)** Partial regression plot illustrating the correlation between [^18^F]PBR111 DVR in the hippocampus and BDI scores in patients with multiple sclerosis (*n* = 11) after correcting for age, duration of disease, and Expanded Disability Status Scale scores. Values represent standardized residuals of the dependent variable (BDI scores) and [^18^F]PBR111 DVR when both variables are regressed on the rest of the independent variables (age, duration of disease, and Expanded Disability Status Scale scores). The BDI scores are represented on the ordinate, and [^18^F]PBR111 DVR is represented on the abscissa.

A current diagnosis of MDE was associated with a higher hippocampal [^18^F]PBR111 DVR among patients with MS, relative to patients with no history or with recent but resolved MDE (current MDE, 1.11 ± .018; no history and resolved MDE [pooled together], 1.06 ± .047; *t* = 2.48, *p* = .044) (Figure 2A). The hippocampal [^18^F]PBR111 DVR values of the three patients with MS and no history of MDE diagnosis (range, 1.03–1.06) were within the 95% confidence interval for the mean of [^18^F]PBR111 DVR of healthy control subjects (95% confidence interval = .98–1.07).

There was a positive correlation between [^18^F]PBR111 DVR in the hippocampus and BDI (Pearson *r* = .63, *p* = .037) in patients with MS. The strength of the independent relationship was stronger when controlled for age, disease duration, and EDSS scores (Pearson partial correlation *r* = .863, *p* = .006) (Figure 2B). We did not find a similar significant association between [^18^F]PBR111 V_T_ and BDI (Pearson partial correlation *r* = −.119, *p* = .778). Correlations between individual BDI items or symptoms clusters and hippocampal [^18^F]PBR111 are displayed in Appendix A, Appendix A. No correlation was found between [^18^F]PBR111 DVR in the hippocampus and EDSS scores, even after controlling for age and disease duration. Similarly, no correlation was observed between [^18^F]PBR111 DVR in the hippocampus and Fatigue Severity Scale scores, and the correlation between hippocampal [^18^F]PBR111 DVR and BDI remained significant with additional correction for the Fatigue Severity Scale.

We did not find a significant correlation between BDI and [^18^F]PBR111 DVR in the thalamus (Pearson *r* = .23, *p* = .58) or with the neocortical V_T_ (Pearson *r* = −.23, *p* = .57) after controlling for age, disease duration, and EDSS. For patients with MS, a linear regression model with age, disease duration, and EDSS explained 48% of the BDI variance, whereas inclusion of hippocampal [^18^F]PBR111 DVR increased the BDI variance explained by the model to 82%. The [^18^F]PBR111 DVR alone explained 33% of the BDI variance. Appendix A, Appendix A illustrates the strength of association of each individual regressor to BDI in patients with MS.

### Hippocampal Resting-State Functional Connectivity

**Figure 3:**
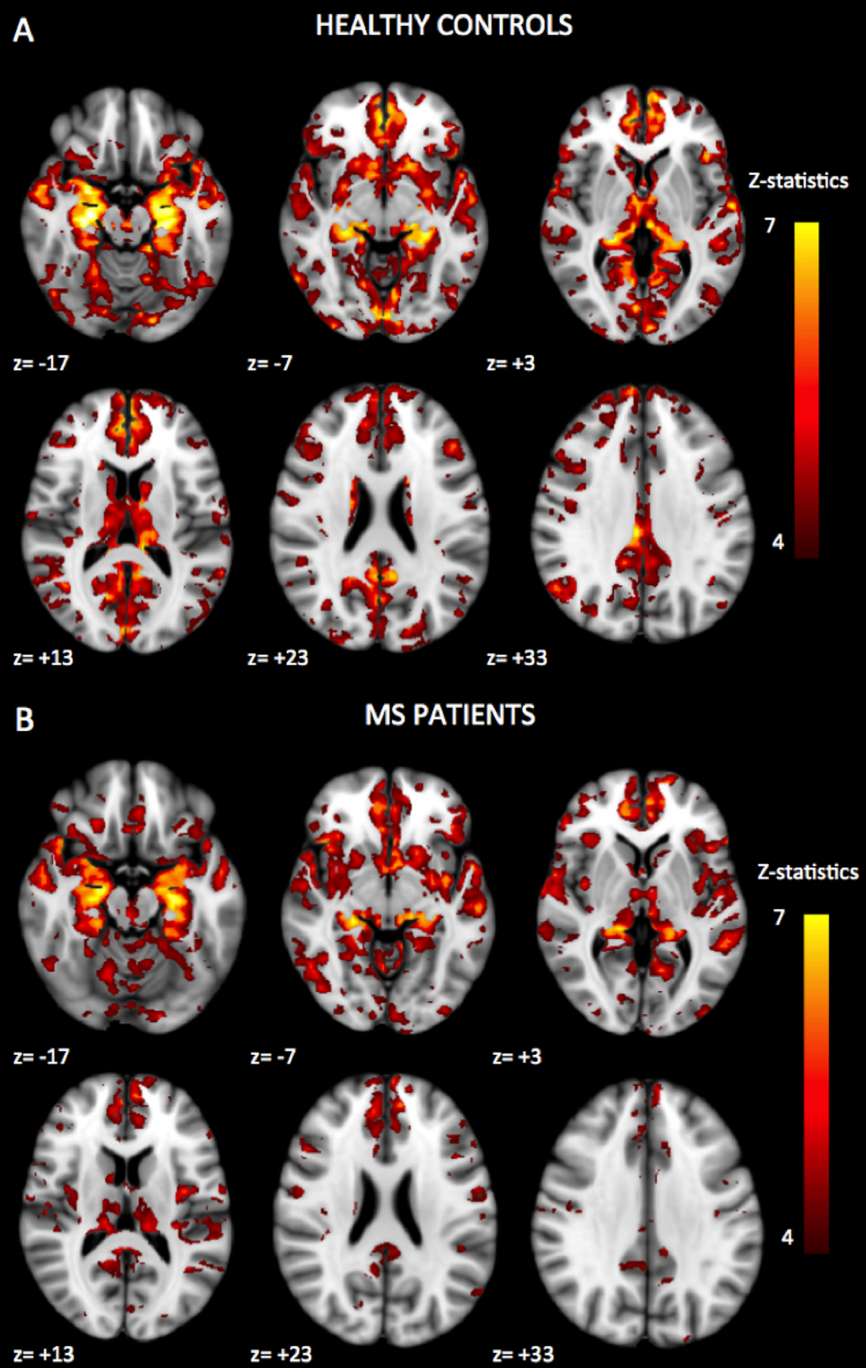
Patterns of significant positive functional connectivity with the hippocampal seed in healthy control subjects and patients with multiple sclerosis (MS). The threshold was set at *z* = 4, *p* < .05 (cluster-corrected for multiple comparisons). The right side of the brain is to the left in the figure (images in radiologic orientation). The regions showing significant positive functional connectivity with the hippocampal seed were located in the limbic system (parahippocampal gyrus, amygdala, and insula), basal ganglia (putamen and caudate), thalamus, temporal lobe (superior, middle, and inferior temporal gyri), medial and inferior prefrontal cortex (anterior cingulate cortex, subgenual cingulate, inferior frontal gyrus, and orbital gyrus), parietal lobe (posterior cingulate, precuneus, angular gyrus, and postcentral gyrus), occipital lobe, and cerebellum. A similar pattern of functional connectivity was found in the patients with multiple sclerosis.

Whole-brain statistical contrasts of hippocampal functional connectivity did not reveal significant group differences between patients with MS and healthy control subjects. Patterns of hippocampal functional connectivity in patients with MS and healthy control subjects are illustrated in Figure 3.

**Figure 4:**
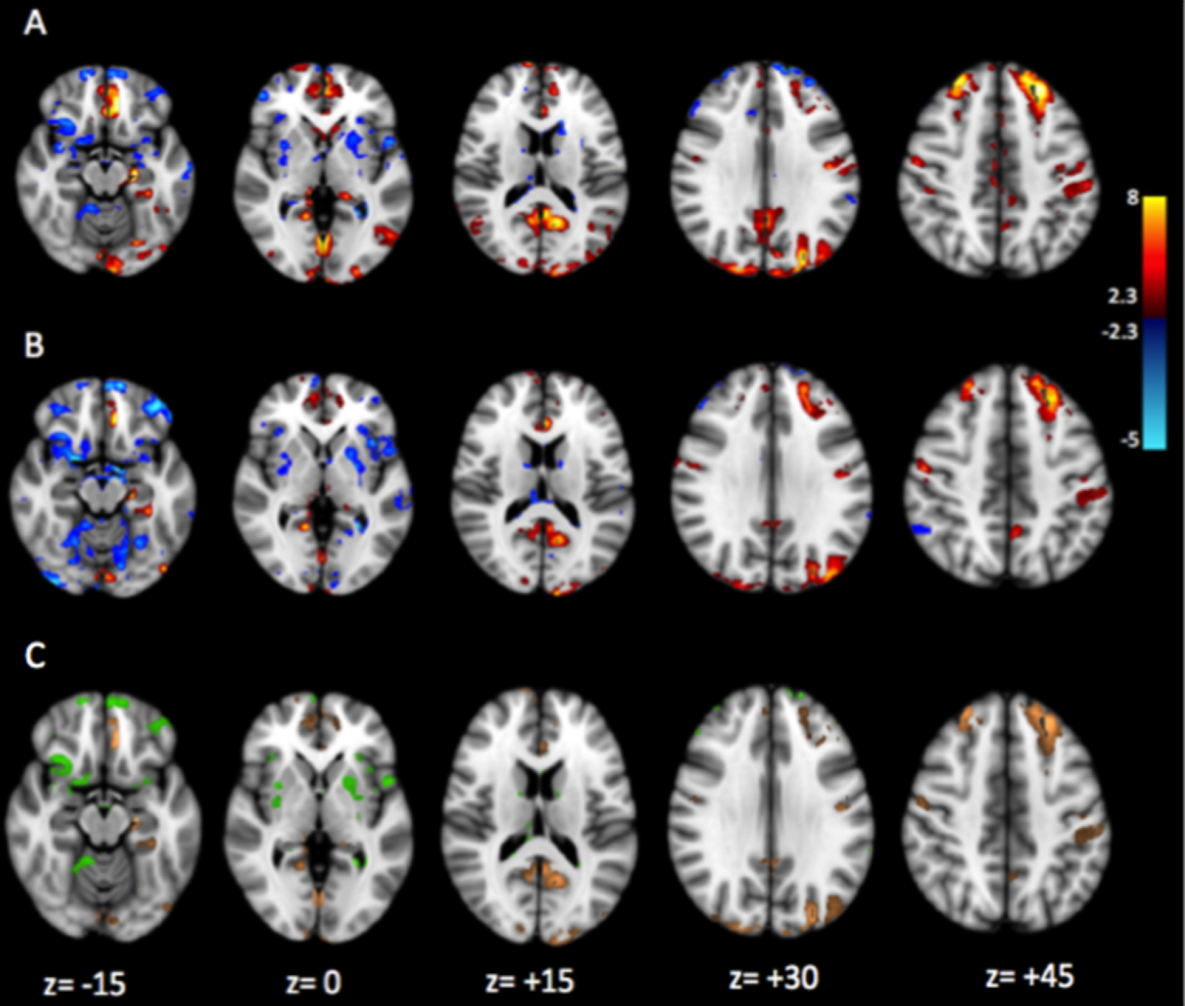
Whole-brain map of correlations of hippocampal functional connectivity to hippocampal [^18^F]PBR111 binding and depressive symptoms in patients with multiple sclerosis. **(A)** Loci with significant correlation between [^18^F]PBR111 distribution volume ratio and strength of functional connectivity to the hippocampus. **(B)** Loci with significant correlation between Beck Depression Inventory scores and strength of functional connectivity to the hippocampus. The threshold was set at *z* = 2.3, *p* = .05 (cluster-corrected for multiple comparisons). Loci with positive correlations are displayed in red-to-yellow, and loci with negative correlations are displayed in blue-to-cyan. **(C)** Conjunction map illustrating loci with significant correlations between strength of hippocampal functional connectivity and both [^18^F]PBR111 distribution volume ratio and Beck Depression Inventory scores. Loci with positive correlations are displayed in orange, and loci with negative correlations are displayed in green.

Hippocampal [^18^F]PBR111 DVR in patients with MS was positively correlated with the strength of functional connectivity between hippocampus and regions in the prefrontal (cingulate cortex and inferior frontal, orbital, and precentral gyri), parietal (posterior cingulate, precuneus, and angular and postcentral gyri), and occipital cortices (*p* < .05 or *z* = 2.3; cluster-corrected for multiple comparisons) (Figure 4A). Negative correlations were found with functional connectivity to the insula, temporal lobe, and basal ganglia. In healthy control subjects, the pattern of correlations of hippocampal [^18^F]PBR111 DVR to functional connectivity of the hippocampus was different. Small clusters of positive correlations were observable in the operculum cortex, whereas negative correlations could be seen in the anterior cingulate gyrus (Appendix A, Appendix A).

The BDI scores in patients with MS were positively correlated with functional connectivity between hippocampus and the medial prefrontal cortex; cingulate cortex; subgenual, orbital, parahippocampal, and lingual gyri; posterior cingulate; precuneus; and occipital cortex (*p* < .05 or *z* = 2.3; cluster-corrected for multiple comparisons) (Figure 4B). Negative correlations were observed in the frontal pole, frontal orbital cortex, putamen, insula, middle temporal gyrus, and lingual gyrus.

**Figure 5:**
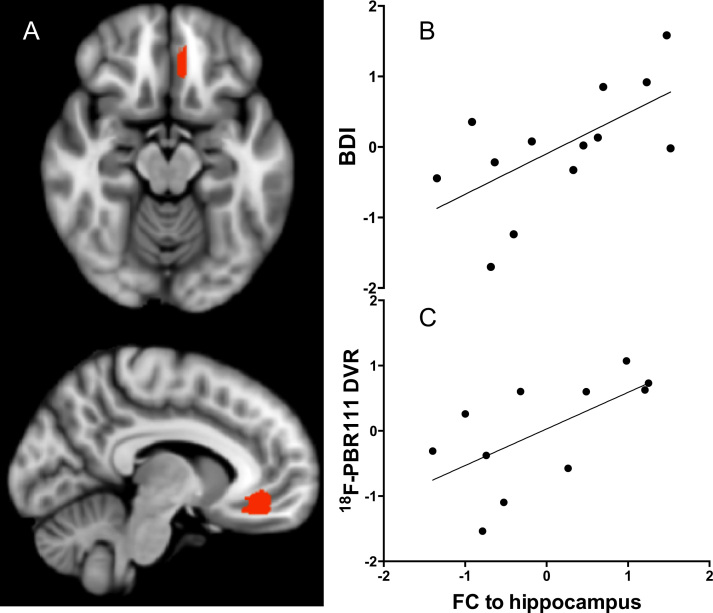
Relationship between hippocampal functional connectivity (FC), hippocampal [^18^F]PBR111 distribution volume ratio (DVR), and Beck Depression Inventory (BDI) scores. **(A)** An area of the prefrontal cortex that included the subgenual cingulate and orbital gyrus (Brodmann areas 11, 12, 25) was chosen from the conjunction map in Figure 4C for illustration purposes. **(B, C)** Scatterplots illustrate the high correlation of the three variables, hippocampal FC, BDI scores, and hippocampal [^18^F]PBR111 DVR, to each other. Values represent standardized residuals of the dependent variables (BDI scores and [^18^F]PBR111 DVR, respectively) and standardized residuals of FC of the hippocampus when all these variables are regressed on the rest of the independent variables (age, duration of disease, and Expanded Disability Status Scale scores).

Correlations of hippocampal functional connectivities with [^18^F]PBR111 DVR and with BDI scores showed moderate overlap (Jaccard index = .34) (Figure 4C). Brain volumes with positive correlations for both were localized in the frontal lobe (subgenual and anterior cingulate cortex and superior and middle frontal gyri), parietal lobe (precuneus and posterior cingulate), and occipital lobe. Brain volumes with negative correlations included insula, frontal lobe areas, and putamen. In a post hoc analysis, we characterized correlations of hippocampal functional connectivity to a prefrontal volume that included the subgenual cingulate and orbital gyrus [selected on the basis of their reported functional association with symptoms of depression in earlier work (34)] with both the radioligand uptake and the BDI (Figure 5A). We found a strong correlation for both measures (Pearson partial correlations *r* = .63 for [^18^F]PBR111 DVR and *r* = .62 for BDI) (Figure 5B, C).

## Discussion

We observed a higher [^18^F]PBR111 binding in the hippocampus of patients with MS relative to healthy control subjects, consistent with previous data identifying a substantial inflammatory disease burden in the hippocampus of patients with MS (11, 12). In our exploratory association analyses, we found that hippocampal [^18^F]PBR111 binding in patients with MS was highly correlated to BDI scores and was highest in patients meeting the criteria for current MDE. A significant association between depressive symptoms and [^18^F]PBR111 binding was specific for the hippocampus and not found for other brain regions examined (e.g., the thalamus or in the neocortex generally). Previous observations implicated the hippocampus specifically as a candidate region for the action of microglia-produced proinflammatory cytokines in mediating depressive symptoms. The hippocampus has the highest interleukin-1 receptor density (10), and lipopolysaccharide-induced depressive-like behavior is associated with abnormal cellular activity in the hippocampus (35). Depressive symptoms have been previously associated with structural and functional hippocampal changes in patients with MS (14, 15, 16, 36, 37).

A role for neuroinflammation in the pathogenesis of depression has been postulated more generally (38, 39). Recent TSPO PET imaging studies provided accumulating in vivo evidence to support the association between brain neuroinflammation and depression. Findings include an increased hippocampal [^18^F]PBR111 binding in patients with MS and MDE (present study), globally increased [^18^F]FEPPA V_T_ in patients with depression during a MDE (40), and an increased hippocampal [^11^C](R)-PK11195 binding in patients with euthymic bipolar disorder (41). In contrast, Hannestad *et al.* (42) did not find differences in [^11^C]PBR28 uptake between patients with MDE and healthy control subjects. However, differences in the study populations could account for discrepant findings, as the study by Hannestad *et al.* included only subjects with low markers of peripheral inflammation, and some of their patients with MDE presented with lower depression indices.

Findings have described different anatomic distribution for observed increases in TSPO binding. Although our study and that of Haarman *et al.* (41) found the increases to be restricted to the hippocampus, the study by Setiawan *et al.* (40) in patients with MDE (without other comorbid disease) described an association with global increases in TSPO binding. This finding could suggest differences in neuroinflammatory processes underlying MDE in individuals with and without MS; however, the study by Setiawan *et al.* does not rule out a particular impact of hippocampal inflammation, although it may not have been able to be identified specifically given the strong global correlations that seemed to be present in brains of subjects in the study. However, both study populations were small. This question needs further study.

We explored the mechanistic basis for an association between hippocampal neuroinflammation and depressive symptoms in patients with MS. It has been suggested that intact functional brain connectivity is important to maintain an optimal affective function and that abnormal connectivities underlie aspects of emotional dysregulation observed in depression (43). Therefore, we evaluated the relationship of BDI and [^18^F]PBR111 DVR variation with resting-state functional connectivity of the hippocampus. The hippocampus is anatomically connected to the parahippocampal gyrus, amygdala, prefrontal cortex, thalamus, and basal ganglia (44, 45). The pattern of functional connectivity that we defined is consistent with the anatomic connectivity as well as with previous studies of functional connectivity of the hippocampus (46, 47, 48, 49). We did not observe significant differences in functional connectivity between healthy subjects and patients with MS as previously reported (50), but our study was not optimally powered to detect this, particularly given the potential interactions of functional connectivity with treatment (51, 52).

The neuroanatomic conjunction of correlations of hippocampal functional connectivity with BDI or [^18^F]PBR111 DVR was striking. We interpret this as evidence in support of the hypothesis that microglial activation in patients with MS is a contributing cause of depression, the symptoms of which are associated with altered hippocampal functional connectivity. Pathologic and preclinical evidence supports the hypothesis that neuroinflammation alters hippocampal function and may be a proximate cause of depression. Human postmortem studies in the brain of patients with MS demonstrated that demyelination in the hippocampus is associated with synaptic alterations (53) and that microglia mediate the synaptic degradation via activation of the complement system (54). In EAE rodents, hippocampal neuroinflammation alters adult neurogenesis (17) and causes alterations of gamma-aminobutyric acidergic transmission (55). This effect is likely to be mediated by activated microglia, as incubation of hippocampal slices from healthy mice with activated microglia causes similar alterations of gamma-aminobutyric acidergic transmission (55). This observation may be relevant for depression, considering the similarities of the EAE-associated behavioral syndrome to depressive conditions (56). However, the role of hippocampal neurogenesis in the pathophysiology of depression has not been well characterized yet; extrapolation of findings from animal studies must be done with caution (18, 57).

Theoretical models of depression support the anatomic distribution of differences in hippocampal functional connectivity that we found associated with BDI. Neuroimaging, neuropathologic, and lesion analysis data all implicate the hippocampus in an extended anatomic network formed by the neural projections of the subgenual cingulate and other areas of the medial orbitofrontal cortex, together with other regions such as amygdala, posterior cingulate cortex, ventral striatum, and thalamus. Impaired functions of key nodes within this network could dysregulate emotional expression and give rise to the clinical signs and symptoms of depression (58, 59).

Our study provides important new data but has some limitations. The cross-sectional nature of the study prevents us from making confident inferences of causality. For example, it is impossible to establish whether hippocampal microglia activation causes depressive symptoms or vice versa. We cannot exclude the latter possibility; for example, exposure of rodents to chronic stress and social isolation has been shown to cause proliferation, activation, and priming of hippocampal microglia (60). It is possible that patients with MS, who are in an immunologically “primed” state (61), may be more likely to manifest such a response. However, the apparent anatomic specificity of the microglial response associated with BDI is striking. This important question requires further longitudinal study controlling for effects of disease-modifying treatment and antidepressants.

The use of a normalized measure of [^18^F]PBR111 binding, the DVR, reduces the variability in modeling the brain signal that arises from imprecision in measurement of blood concentrations and hence improves the signal-to-noise ratio. By doing so, the DVR expresses only a relative signal and is appropriate only when testing for differences in the regional distribution of activated microglia. The elimination of global differences in the [^18^F]PBR111 specific signal also reduces the between-subject variability associated with the rs6971 polymorphism of the TSPO gene (32). However, the use of a region that is recognized to bind TSPO radiotracers and in which MS-associated increases in binding are observed must be taken into account when assessing the magnitude and sign of the DVR for a ROI. We cannot exclude that the observed between-groups difference could have been driven by a lower [^18^F]PBR111 binding in the cortical gray matter (the pseudo-reference region) in patients with MS, although this direction of effects (decreased microglial activation is associated with depression) would be counter to expectations based on other observations (3, 39, 40). Moreover, cortical gray matter [^18^F]PBR111 V_T_ as well as [^18^F]PBR111 V_T_ and DVR in all ROIs other than the hippocampus were similar across groups.

A fixed-effects model was used for correlation analysis with functional connectivity measures because of the small sample size. This method is appropriate for examination of effects in samples of particular patient groups, as used here, but it limits the generalization of findings to the whole population, and therefore the extrapolation of our findings to the wider MS population must be made cautiously (62). Our small sample size precluded exploration of the impacts of potential confounding factors such as sex, use of concomitant medications, global and regional brain atrophy, localization of lesions, and alteration in structural connectivity in patients. We observed that overall greater T2 fluid attenuated inversion recovery activity was related to decreased severity of depression. Despite this observation, BDI correlated positively with hippocampal DVR, suggesting a specific association of depression with PET measures of neuroinflammation. Our interest was focused on hippocampal functional connectivity because of the prominence of the microglial activation signal in this region, but it cannot be excluded that hippocampal neuroinflammation is associated with alterations in functional connectivity in regions other than the hippocampus.

The potential role of confounding factors must be taken into account. Clinical factors such as fatigue and cognitive deficits need to be considered; however, depression in patients with MS is a distinct symptom and is not explained by either fatigue or cognitive impairment. Although depressive symptoms were correlated to the level of disability measured with the EDSS, the relationship between hippocampal neuroinflammation and the BDI appeared to be independent of the EDSS; we did not observe a relationship between hippocampal [^18^F]PBR111 DVR and EDSS. We found that hippocampal neuroinflammation was correlated preferably to the cognitive, rather than the somatic, components of the BDI.

Most of the patients with MS studied were receiving disease-modifying treatment, which might have an effect, even if indirect, to reduce microglial activation and the range of variation in [^18^F]PBR111 DVR seen in our sample (63). The interaction between disease-modifying therapy, neuroinflammation, and depression is complex, as some disease-modifying therapies, such as interferon-β, might be associated with depression (64). Additionally, most of the patients with prominent depressive symptoms were on antidepressant treatments (five of six patients with current MDE) that could have further confounded the relationship between depressive symptoms and hippocampal neuroinflammation, by influencing the expression of depressive symptoms, the activation of microglia, or both (65). A longitudinal design will be needed in future work to define independent contributions of these factors.

In conclusion, our findings suggest that mediators of innate immunity in the hippocampus play a significant role in the pathophysiology of the affective dysregulation associated with MS. Our results provide novel insight into the relationship between hippocampal pathology and depressive symptoms in MS and, in conjunction with other observations cited in this article, support a pathogenic role of chronic neuroinflammation in the genesis of depression in MS. Control of neuroinflammatory disease processes may be a rational treatment for depression in patients with MS. This paradigm also suggests a possible role for immune-modulating treatment for major depressive disorder in other contexts.

## Figures and Tables

**Table 1:** Clinical and Demographic Characteristics of Study Participants

Case	Age (Years)/Sex	TSPO Gene Group	Disease Duration (Years)	EDSS	BDI	MDE
Healthy Control Subjects						
1	45/F	MAB	—	—	6	None
2	36/F	MAB	—	—	0	None
3	33/F	HAB	—	—	9	None
4	52/F	LAB	—	—	0	None
5	61/F	MAB	—	—	0	None
6	42/F	HAB	—	—	0	None
7	52/F	HAB	—	—	1	None
8	43/M	MAB	—	—	0	None
9	50/F	HAB	—	—	4	None
10	28/M	HAB	—	—	1	None
11	52/F	HAB	—	—	0	None
12	51/M	LAB	—	—	0	None
13	65/M	LAB	—	—	8	None
14	28/M	HAB	—	—	2	None
15	57/M	MAB	—	—	—	None
16	59/F	HAB	—	—	—	None
17	59/M	HAB	—	—	—	None
18	62/M	HAB	—	—	—	None
19	60/F	MAB	—	—	—	None
20	60/F	MAB	—	—	—	None
21	56/F	MAB	—	—	—	None
22	44/F	LAB	—	—	—	None
Patients With MS		—	—	—	—	—
1	48/F	HAB	8	6.5	23	Current
2	39/F	LAB	20	4	9	Recent
3	40/F	HAB	11	4	24	Current
4	55/F	HAB	20	2	5	None
5	53/F	LAB	20	7	30	Current
6	50/F	HAB	2	4	10	Current
7	59/F	MAB	16	3	8	Recent
8^a^	42/M	MAB	11	5.5	20	Current
9	41/F	HAB	14	1.5	3	None
10	28/M	HAB	7	2	14	Recent
11	41/F	MAB	4	5.5	7	None
12	42/F	HAB	1.5	6	19	Current
13^a^	37/F	—	9	7	23	Current

BDI, Beck Depression Inventory; EDSS, Expanded Disability Status Scale; HAB, high-affinity binder; LAB, low-affinity binder; MAB, mixed-affinity binder; MDE, major depressive episode; TSPO, 18-kDa translocator protein.

a These patients were not included in the positron emission tomography analysis.

## Footnotes

Appendix A Supplementary material cited in this article is available online at http://dx.doi.org/10.1016/j.biopsych.2015.11.022.
