# Supplementary material for: Hippocampal Neuroinflammation, Functional Connectivity, and Depressive Symptoms in Multiple Sclerosis
Source: Biol Psychiatry. 2016 Jul 1;80(1):62–72. doi: 10.1016/j.biopsych.2015.11.022 (PMC4918731; doi:10.1016/j.biopsych.2015.11.022)
Supplement: Supplementary file 1 — Supplementary Material [file mmc1.pdf]

Hippocampal Neuroinflammation, Functional Connectivity and Depressive Symptoms in Multiple Sclerosis

Supplemental Information

Supplemental Methods & Materials

Subjects Recruitment and Screening Procedures

Healthy controls were recruited from a database of volunteers. For healthy volunteers, exclusion criteria included any medical, neurological, or psychiatric illness at screening or any concomitant pharmacological treatments. All structural magnetic resonance imaging (MRI) images were inspected by an experienced neuroradiologist for unexpected findings of potential clinical significance or features that might confound imaging analysis.

Patients with relapsing remitting multiple sclerosis (MS) were referred by consultant neurologists in the Imperial College Healthcare NHS Trust and the University College London Hospitals NHS Foundation Trust. None had received steroid treatment in the month before the positron emission tomography (PET) scans and all had been stable on their medications for at least 3 months prior to scanning. Clinical characteristics of MS patients are described in Table S1.

At baseline screening for eligibility, all study subjects underwent a full medical, neurological and psychiatric examination. During the screening examination, blood was collected for genotyping for the rs6971 polymorphism of the *TSPO* gene. The subjects returned to the research center for the combined MRI and ^{18}F-PBR111 PET scan visit within approximately one week of the screening visit.

All subjects provided written, informed consent prior to enrollment. Expenses of all subjects were reimbursed and they were provided with honoraria recognizing the time and effort of participation.

PET Radioligand Synthesis

[^{18}F]-PBR111 was labeled with fluorine-18 by a simple one-step tosyloxy-for-fluorine nucleophilic aliphatic substitution, followed by purification by semi-preparative high-performance liquid chromatography (HPLC) and reformulation. The synthetic procedure of [^{18}F]-PBR111 was adapted from a previously described method (1). A fully automated procedure was developed in-house using a Siemens Explora GN module coupled with a semi-preparative HPLC system.

[^{18}F]-Fluoride, produced using a Siemens RDS-111 Eclipse cyclotron equipped with a fluoride target loaded with oxygen-18 enriched water, was obtained by means of the ^{18}O(p,n)^{18}F reaction. The [^{18}F]-fluoride in solution in oxygen-18 enriched water was then trapped onto a DW-TRC-L trap and release cartridge (O.R.T.G.). Following that first step the fluoride was released into the reactor using 1.0 mL of a solution consisting of 8.3 mL of acetonitrile, 0.8 mL of water, 250 mg of K_{222} and 50 mg of K_2CO_3. The content of the reactor was evaporated a first time and then the evaporation process was repeated 2 times following the addition of 1 mL of acetonitrile each time. The toxyloxy precursor (6.0-10.0 mg in solution in 1.0 mL of anhydrous acetonitrile) was then added to the reactor containing the "dry" [^{18}F]-fluoride. The reaction mixture was heated for 5 min at 100ºC and then cooled to 50ºC before diluting with water. The crude reaction mixture was then diluted with water (3 ml) and loaded onto the HPLC injection loop for purification on an Agilent Eclipse XDB C18 column (5 µm, 250 × 9.4 mm) with

ammonium formate buffer (pH 4)/CH_3CN (55:45, v/v) at 9.5 mL/min. The fraction containing [^{18}F]-PBR111 was collected in water (20 ml) and loaded onto an activated Waters Sep-Pak® Classic C18 cartridge (Waters Corp.) for reformulation. Following an initial SepPak® column wash with water, [^{18}F]-PBR111 was eluted off the column with ethanol and 0.9% saline solution for injection to lead to [^{18}F]-PBR111 formulated in 11 mL of maximum 20% (v/v) ethanol in 0.9% saline solution for injection. In the final step a sterile filtration through a 0.2 µm sterile filter (Millipore Millex GV, 0.22 µm, 33 mm) was performed to deliver the final dose as a sterile and pyrogen-free solution.

Typically, the total [^{18}F]-PBR111 synthesis procedure, including HPLC purification and Sep-Pak®-based formulation, was accomplished in less than 60 min. Up to 4.2 GBq of [^{18}F]-PBR111 (>97% radiochemically pure, n > 20) were obtained starting from 10 GBq of [^{18}F]-fluoride with a specific radioactivity of up to 480 GBq/µmol.

PET Protocol

[^{18}F]-PBR111 was injected as an intravenous bolus over approximately 20 seconds and PET data collected in 3D-mode for 120 min post injection. The injected mass and radioactivity of [^{18}F]-PBR111 were similar for healthy controls and MS patients [healthy controls, mass 0.32 (0.14 – 8.66) µg [median (range)], radioactivity 166 (143 – 182) MBq; MS patients, mass 0.49 (0.2 – 1.9) µg, radioactivity 166 (150-179) MBq].

PET data were reconstructed using filtered back projection including corrections for attenuation and scatter (based on a low-dose computerized tomography acquisition). Dynamic data were binned into 29 frames (durations: 8 x 15 s, 3 x 1 min, 5 x 2 min, 5 x 5 min, 8 x 10 min).

Arterial blood data were sampled via the radial artery to enable generation of an arterial plasma input function. A continuous sampling system (ABSS Allogg, Mariefred, Sweden) was used to measure whole blood activity each second for the first 15 minutes of each scan. Discrete blood samples were manually withdrawn at 5, 10, 15, 20, 25, 30, 40, 50, 60, 70, 80, 90, 100, 110 and 120 minutes after scan start to facilitate measurement of whole blood and plasma activity. Samples taken at 5, 10, 15, 20, 30, 50, 70, 90 and 120 minute time points were also analyzed using HPLC to determine the fraction of radioactivity corresponding to intact parent compound in arterial plasma. The first three discrete blood samples were used to calibrate the continuous blood data, and then the continuous and discrete data sets were used to form a whole blood activity curve covering the duration of the scan. Discrete plasma samples were divided by the corresponding whole blood samples to form plasma-over-blood (POB) data. A constant POB model was fitted (i.e., average of POB values). This POB value was then multiplied by the whole blood curve to generate a total plasma curve. Parent fraction data were fitted to a sigmoid model $f = ((1-(t^3/(t^3+10^a)))^b+c)/(1+c)$ where t is time and a, b and c are fitted parameters. The resulting fitted parent fraction profile was multiplied by the total plasma curve and then smoothed post-peak using a tri-exponential fit to derive the required parent plasma input function. For each scan, a time delay was fitted and applied to the input function to account for any temporal delay between blood sample measurement and the tomographic measurements of the tissue data.

Definition of Regions of Interest

The hippocampus and thalamus were chosen a priori. The post-hoc exploratory analysis included cortical gray matter regions of interest (ROIs) (frontal cortex, occipital cortex, parietal cortex,

temporal cortex, cingulate cortex, insula), as well as caudate, putamen, cerebellum, midbrain, pons and medulla.

Due to defluorination of the metabolite (1) [^{18}F]-PBR111 defluorinates in vivo resulting in a relatively high signal in the bones of the skull (2, 3). The ROIs for the cortical gray matter reference region, as well as for the frontal, occipital, parietal, temporal, cingulate, and insular cortices, were therefore eroded by 5 mm away from the skull to avoid “spill-in” from uptake of [^{18}F]-fluoride by bone of the skull. All the ROIs were then applied to the dynamic PET data to derive regional time activity curves.

The pre-chosen ROIs and the cortical gray matter were defined with the MNI152 template (http://www2.bic.mni.mcgill.ca), derived from an average of MRI brain scans from the Montreal Neurological Institute. The template was nonlinearly warped to the high-resolution T1-MRI of each individual, with SPM5 (Statistical Parametric Mapping; Wellcome Trust Centre for Neuroimaging). The deformation parameters derived were then applied to a corresponding anatomical atlas, the CIC (Clinical Imaging Center) atlas (4) to bring this into the space of the individual subject. Finally, the MRI image, ROIs, and warped anatomical atlas were resampled to match the PET image resolution.

Quantification of TSPO Signal

The [^{18}F]-PBR111 total volume of distribution (V_T) was quantified using a 2-tissue compartment model with metabolite-corrected arterial input function and a fixed blood volume correction of 5%, as previously described (3). For each ROI examined, the total V_T was estimated from the rate constants as described previously (5). The Logan graphical method (6) employing a plasma input, using a fixed blood volume correction of 5% and a linear start time at 35 minutes was also

used to estimate the V_T of each ROI and further applied at the voxel level to produce parametric V_T maps. Model fitting and parameter estimation were performed using software implemented in MATLAB R2008b (The MathWorks, Inc.).

MRI Protocol

MRI data was acquired on a 3T Siemens Verio (Siemens Healthcare, Erlangen Germany) clinical MRI scanner, equipped with a 32-channel phased-array head coil. T1-weighted structural images were applied for PET co-registration and used the Alzheimer’s Disease Neuroimaging Initiative recommended parameters with a parallel imaging factor of 2 (7). T1-weighted images were acquired pre- and post-iv gadolinium-chelate administration (0.1 mmol/kg gadoteric acid, Dotarem®).

Volumetric T2-weighted FLAIR images were acquired using a 1mm isotropic resolution 3D SPACE sequence (8), with a 250 x 250 x 160 mm FOV, TE = 395 ms, TR = 5 s, TI = 1800 ms, turbo factor of 141, 256 x 256 x 160 matrix, and parallel imaging factor of 2 in 5 m: 52 s.

Resting-state fMRI data was acquired with a double-echo gradient echo EPI sequence, capturing 150 volumes in 5 m: 06 s. Each volume comprised 35 3-mm thick slices in a 225 mm FOV, 64 x 64 matrix size, echo times at 13 and 31ms, parallel imaging factor of 2, and a TR of 2 seconds.

Statistical Analyses

Statistical analyses were done using SPSS 20. Analyses were divided into group analyses, exploratory association analyses and functional connectivity analysis.

Group Analyses

We used *t*-tests for independent samples for between-groups comparisons of age and Beck Depression Inventory (BDI) scores, while gender distribution was studied using a Chi-squared test. For [18F]-PBR111 distribution volume ratio (DVR) in the hippocampal and thalamic ROI and V_T in the cortical gray matter, we used both a *t*-test and an analysis of covariance (ANCOVA) with DVR or V_T as dependent variables, group (or diagnosis of major depressive episode) and rs6971 genotype as fixed factors, and age as covariate. A post-hoc analysis of group differences in all major gray matter ROIs was performed using only the ANCOVA.

Exploratory Association Analyses

The correlation between BDI scores, age, Expanded Disability Status Scale (EDSS) scores, and disease duration in patients with MS was studied using Pearson's correlation. The correlation between hippocampal [18F]-PBR111 DVR and BDI scores was exclusively studied in patients with MS using Pearson's partial correlation, while controlling for age, EDSS scores, and disease duration (i.e., the same nuisance variables as used in the model to study correlations of functional connectivity to depressive symptoms and PET signal). The choice of these nuisance variables was defined a priori based on their potential effect on the relationship between the two main variables of interest, namely depressive symptoms scores and hippocampal neuroinflammation. Age is known to moderate the relationship of physical dysfunction to psychological distress in MS patients (9) while EDSS and disease duration have been found associated with TSPO PET signal in earlier PET studies in MS (10, 11), and have also been found to be associated to MS depression in previous epidemiological reports (12).

Correlation and partial-correlation analysis of BDI were performed between hippocampal [18F]-PBR111 DVR and subtotal scores of the cognitive and somatic clusters as well as

individual scores of BDI items.

Two linear regression models were explored in order to study the proportion of variance in BDI scores that would be explained by hippocampal [^{18}F]-PBR111 binding: first, we tested a model with BDI scores as the dependent variable and age, disease duration, and EDSS as explanatory variables. Then, we subsequently added to the model hippocampal [^{18}F]-PBR111 DVR as an additional explanatory variable. Statistical significance for all analyses was set at the $p < 0.05$ level.

Both [^{18}F]-PBR111 DVR and BDI data were normally distributed as determined by visual inspection of Q-Q plots and Shapiro-Wilk test for normality.

MRI and Functional Connectivity Analysis

Each subject's T1 anatomical images were stripped of their non-brain tissue using the brain extraction tool (BET) v2.1 (fractional intensity threshold = 0.5, robust brain center estimation). A bilateral hippocampal ROI was defined in standardized stereotactic (MNI152) space. The entire hippocampus was defined using a standard anatomical atlas (the CIC Atlas, thresholded at 0.5). This template hippocampal region was then registered into each subject's functional data space using FLIRT (FSL's Linear Registration Tool). Time-series data were then extracted from the functional data using the transformed subject-specific hippocampal ROI as a mask image.

The fMRI Expert Analysis Tool (FEAT) v6.00 was used for pre-processing, general linear model analysis and anatomical registration of the fMRI data. Pre-processing included temporal high-pass filtering (0.01 Hz), head-motion correction, and spatial smoothing with a 5 mm full-width half-maximum Gaussian kernel. First-level analyses were conducted on each individual subject/scan by entering the extracted hippocampal time-course as a regressor,

together with head-motion parameters as regressors of no interest. No haemodynamic modeling was added to the hippocampal regressor, as it already represents haemodynamic data. Contrasts simply modeled the positive and negative effects of the hippocampal time-series regressor. The resulting statistical images were registered to the subject's main anatomical image (acquired during the same session) and subsequently non-linearly warped to the template T1 anatomical image in standard MNI-space.

Group-level analyses were also conducted using FEAT, and used a mixed-model (FLAME-1) approach to test between-groups differences in order to model both within and between-subject variance. Age and gender were included in this model as additional regressors of no interest. The analysis of correlations of hippocampal functional connectivity to hippocampal [^{18}F]-PBR111 DVR was done separately for MS patients and healthy controls. [^{18}F]-PBR111 DVR was the regressor of interest in the group-level model, while nuisance regressors included age, disease duration, and EDSS for MS patients, and age for healthy controls. The analysis of correlations of hippocampal functional connectivity to depressive symptoms was performed only on MS patients' data. For this analysis BDI was included as regressor of interest and nuisance regressors included age, disease duration, and EDSS. In order to maximize sensitivity, fixed-effects models were used for the analysis of correlations.

The Jaccard index was used to quantify the overlap between correlation maps of hippocampal functional connectivity to [^{18}F]-PBR111 DVR and BDI respectively, and was computed as follows:

(1) $$J(A,B) = \frac{|A \cap B|}{|A \cup B|}$$

where A is the map of loci with significant correlations (positive and negative) between hippocampal functional connectivity and $[^{18}F]$-PBR111 DVR, and B is the map of loci with significant correlations between hippocampal functional connectivity and BDI.

Brain tissue volume, normalized for subject head size, and hippocampal volume were estimated respectively with SIENAX (13) and FIRST (14), part of FSL (15). T2-FLAIR hyperintense WM volumes were segmented using JIM version 6.0 (Xinapse Systems, Northants).

Supplemental Results

Exploratory Group Analyses

We found no significant group differences in either $[^{18}F]$-PBR111 V_T or DVR in any ROIs other than the hippocampus, in a post-hoc exploratory test of all major ROIs (Table S2).

An analysis using a subgroup of healthy subjects (n = 11), matched for TSPO binding status and age to the MS patients group, also demonstrated a higher $[^{18}F]$-PBR111 DVR in the hippocampus of MS patients relative to controls ($t = 2.891$; $p = 0.009$), while all V_T or DVR values of the other ROIs did not differ between groups (Table S3).

T2 FLAIR Lesions and Brain Volumes

No T2 FLAIR or gadolinium-enhancing lesions were found in the hippocampus of subjects. We observed a negative correlation between T2 FLAIR volumes and hippocampal DVR ($r = -0.65$; $p < 0.05$), while there was no relationship between T2 FLAIR volumes and cortical or hippocampal V_T. T2 FLAIR volumes were not associated to EDSS, while they were negatively correlated to BDI, and the inverse correlation remained significant after correction for age, disease duration and EDSS ($r = -0.85$; $p < 0.01$).

MS patients had reduced total brain (MS, 1,438 $cm^3 \pm 60$ (mean ± SD); healthy controls, 1,506 $cm^3 \pm 56$; $t = 3.05$; $p = 0.005$) and gray matter (MS, 741 $cm^3 \pm 40$ [mean ± SD]; healthy controls, 786 $mm^3 \pm 37$; $t = 3.06$; $p = 0.005$) volumes compared to healthy controls. The differences remained significant after correction for age and gender (total brain volume: $F = 11.19$; $p = 0.003$; gray matter volume: $F = 8.43$; $p = 0.008$). We did not observe a correlation in MS patients between hippocampal DVR and total brain or gray matter volumes after correction for age, disease duration and EDSS. Similarly, brain volumes were not correlated to cortical V_T even after further correction for TSPO binding group. Total brain and gray matter volumes in MS patients were not significantly associated to EDSS or BDI.

Hippocampal volumes were smaller in MS patients relative to healthy controls (MS, 6,731 $mm^3 \pm 1086$ healthy controls, 7,644 $mm^3 \pm 478$; $t = 2.77$; $p = 0.016$) (mean ± SD) and the difference remained significant after correction for age and gender ($F = 7.97$; $p = 0.008$). Hippocampal volumes were not correlated to hippocampal [^{18}F]-PBR111 DVR, nor to EDSS or BDI. Our main finding of an increased hippocampal [^{18}F]-PBR111 DVR in MS patients, relative to healthy subjects, reached stronger significance with the correction for hippocampal volumes ($F = 7.76$; $p = 0.01$). This observation could indicate that smaller hippocampal volumes may affect the precision of measurement of [^{18}F]-PBR111 binding, by diluting the signal arising from the hippocampus.

Table S1. Clinical characteristics of MS patients

MS Patients Case	T2-FLAIR Lesion Load mm^3	Brain Tissue Volume cm^3	Hippocampal Volume mm^3	Disease Modifying Treatment	Antidepressant Treatment
1	9438	1434	5619	natalizumab	Amitriptyline
2	32088	1417	6439	interferon b	Citalopram
3	1077	1470	7000	natalizumab	Mirtazapine, Citalopram
4	32098	1454	7508	interferon b	-
5	929	1505	7756	-	Citalopram
6	20179	1487	6229	interferon b	-
7	8214	1355	6428	-	-
8	599	1385	4764	interferon b	-
9	8942*	1411	7671	-	-
10	5207	1451	8649	interferon b	Citalopram
11	23331	1364	5693	interferon b	-
12	4677	1571	7023	interferon b	-
13	n/a §	1396	n/a §	interferon b	Citalopram

T2 FLAIR lesions, whole brain and hippocampal volumes, disease-modifying and antidepressant treatment across MS patients.

* presence of gadolinium-enhancing lesions.

§ T2-FLAIR lesions load and hippocampal volumes could not be calculated due to incomplete acquisition of MRI data.

Table S2. Significance of differences in [^{18}F]-PBR111 V_T and DVR between MS and healthy control groups across ROIs

**ROI**	**V_T**		**DVR**	
	F	***p* Values**	***F***	***p* Values**
Frontal cortex	0.01	0.92	3.28	0.08
Occipital cortex	0.03	0.87	1.93	0.18
Parietal cortex	0.00	0.98	0.04	0.85
Temporal cortex	0.01	0.94	0.67	0.42
Cingulate cortex	0.02	0.90	0.06	0.82
Insula	0.01	0.92	0.08	0.78
Cerebellum	0.00	0.96	0.02	0.90
Brainstem	0.00	0.96	0.11	0.75
Striatum	0.02	0.90	0.00	0.97
Thalamus	0.05	0.83	0.66	0.43
Hippocampus	0.25	0.63	5.73	0.02*

Post-hoc exploratory comparison of [^{18}F]-PBR111 V_T and [^{18}F]-PBR111 DVR in all major gray matter ROIs between MS patients and controls.

* The ANCOVA showed a significant group difference only in hippocampal [^{18}F]-PBR111 DVR.

Table S3. Post-hoc analysis of sub-groups matched for age and genotype

		HV		MS		
No. subjects		11		11		
HAB:MAB:LAB		7:2:2		7:2:2		
Gender (M:F)		3:8		1:10		
		**Mean**	**SD**	**Mean**	**SD**	
Age (years)		44.9	11.82	45.1	8.86	
Age HAB group (years)		43.1	13.02	43.4	8.71	
**ROIs**		**Mean**	**SD**	**Mean**	**SD**	***p* Value**
Frontal cortex	V_T	3.29	1.13	2.90	0.96	0.40
	DVR	1.00	0.03	0.98	0.03	0.07
Occipital cortex	V_T	3.42	1.21	3.14	0.97	0.57
	DVR	1.04	0.05	1.07	0.05	0.23
Parietal cortex	V_T	3.17	1.09	2.89	0.92	0.52
	DVR	0.97	0.02	0.98	0.02	0.29
Temporal cortex	V_T	3.36	1.24	3.04	0.96	0.50
	DVR	1.02	0.05	1.03	0.03	0.66
Cingulate cortex	V_T	3.14	1.14	2.92	1.01	0.64
	DVR	0.96	0.06	0.98	0.05	0.31
Insula	V_T	3.13	1.11	2.89	0.96	0.60
	DVR	0.95	0.06	0.97	0.04	0.37
Cerebellum	V_T	3.17	1.48	2.82	0.93	0.52
	DVR	0.94	0.12	0.96	0.07	0.71
Brainstem	V_T	3.57	1.47	3.30	1.17	0.64
	DVR	1.07	0.13	1.11	0.15	0.55
Striatum	V_T	2.96	1.13	2.78	0.98	0.69
	DVR	0.90	0.08	0.93	0.06	0.35
Thalamus	V_T	3.50	1.35	3.23	1.10	0.61
	DVR	1.06	0.07	1.09	0.07	0.39
Hippocampus	V_T	3.25	1.23	3.19	0.99	0.89
	DVR	0.99	0.10	1.08	0.04	0.01*

Tests for between-groups differences in [^{18}F]-PBR111 V_T and [^{18}F]-PBR111 DVR in gray matter ROIs between MS patients and controls matched for age and TSPO binding status.

Table S4. Correlation of hippocampal DVR to individual BDI items

**BDI-II Items**	**Correlation Coefficients (uncorrected)**	**Partial Correlation Coefficients (corrected for age, disease duration, EDSS)**
Sadness [a]	0.79 **	0.84 **
Pessimism [a]	0.12	-0.37
Past failure [a]	0.59 *	0.62
Loss of pleasure [b]	0.31	0.17
Guilty feelings [a]	0.53	0.77 *
Punishment feelings [a]	0.14	0.16
Self-dislike [a]	0.47	0.52
Self-criticalness [a]	0.42	0.50
Suicidal thoughts or wishes [a]	0.38	0.64
Crying [c]	0.50	0.57
Agitation [c]	0.47	0.71 *
Loss of interest [b]	0.19	0.33
Indecisiveness [c]	0.46	0.70 *
Worthlessness [b]	0.58	0.62
Loss of energy [b]	0.53	0.55
Changes in sleeping pattern [b]	0.22	0.09
Irritability [b]	0.39	0.42
Changes in appetite [b]	0.39	0.59
Concentration difficulty [b]	0.33	0.62
Tiredness or fatigue [b]	0.40	0.49
Loss of interest in sex [c]	0.36	0.42
**Cognitive cluster**	0.73 **	0.94 ****
**Somatic cluster**	0.46	0.62

Correlation and partial correlation analyses between individual BDI items and symptoms clusters to hippocampal [^{18}F]-PBR111 DVR.

a cognitive cluster items.

b somatic cluster items.

c items not included in a cluster.

*$p \leq 0.05$.

**$p < 0.01$.

*** $p < 0.005$.

**** $p < 0.0005$.

Table S5. Linear regression analysis

**Regressors**	***b* Coefficient**	***T***	***p* Value**	**Correlation Coefficients**	
				**Zero-order**	**Partial**
EDSS	0.696	4.694	0.003	0.717	0.887
Hippocampal DVR	0.657	4.185	0.006	0.631	0.863
Disease duration	0.460	2.795	0.031	-0.053	0.752
Age	-0.473	-2.895	0.028	-0.002	-0.763

Linear regression analysis model with BDI as dependent variable. The contribution of each individual regressor is indicated.

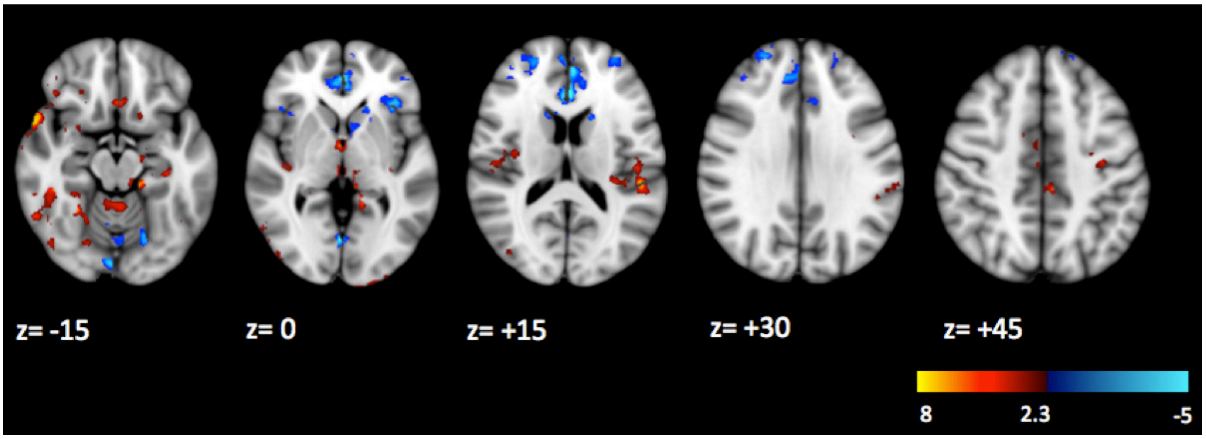

Figure S1. Whole brain map of correlations of hippocampal functional connectivity to hippocampal [^{18}F]-PBR111 binding in healthy volunteers. Loci with significant correlation between [^{18}F]-PBR111 DVR and strength of functional connectivity to the hippocampus in healthy subjects. Threshold was set at Z = 2.3, p = 0.05 (cluster-corrected for multiple comparisons). Loci with positive correlations are displayed in red-to-yellow, loci with negative correlations are displayed in blue-to-cyan.

Supplemental References

1. Fookes CJ, Pham TQ, Mattner F, Greguric I, Loc'h C, Liu X, *et al.* (2008): Synthesis and biological evaluation of substituted [18F]imidazo[1,2-a]pyridines and [18F]pyrazolo[1,5-a]pyrimidines for the study of the peripheral benzodiazepine receptor using positron emission tomography. *Journal of medicinal chemistry*. 51:3700-3712.

2. Carson RE, Wu Y, Lang L, Ma Y, Der MG, Herscovitch P, et al. (2003): Brain uptake of the acid metabolites of F-18-labeled WAY 100635 analogs. *Journal of cerebral blood flow and metabolism*. 23:249-260.

3. Guo Q, Colasanti A, Owen DR, Onega M, Kamalakaran A, Bennacef I, *et al.* (2013): Quantification of the Specific Translocator Protein Signal of [18F]PBR111 in Healthy Humans: A Genetic Polymorphism Effect on In Vivo Binding. *J Nucl Med*. 54:1915-1923.

4. Tziortzi AC, Searle GE, Tzimopoulou S, Salinas C, Beaver JD, Jenkinson M, *et al.* (2011): Imaging dopamine receptors in humans with [11C]-(+)-PHNO: dissection of D3 signal and anatomy. *NeuroImage*. 54:264-277.

5. Gunn RN, Gunn SR, Cunningham VJ (2001): Positron emission tomography compartmental models. *J Cereb Blood Flow Metab*. 21:635-652.

6. Logan J, Fowler JS, Volkow ND, Wolf AP, Dewey SL, Schlyer DJ, *et al.* (1990): Graphical analysis of reversible radioligand binding from time-activity measurements applied to [N-11C-methyl]-(-)-cocaine PET studies in human subjects. *J Cereb Blood Flow Metab*. 10:740-747.

7. Jack CR, Jr., Bernstein MA, Fox NC, Thompson P, Alexander G, Harvey D, *et al.* (2008): The Alzheimer's Disease Neuroimaging Initiative (ADNI): MRI methods. *Journal of magnetic resonance imaging : JMRI*. 27:685-691.

8. Mugler JP, Brookeman JR (2003): Ultra-Long Echo Trains for Rapid 3D T2-Weighted Turbo-Spin-Echo Imaging. *Proceedings of the 11th International Society of Magnetic Resonance in Medicine*.970.

9. Jones SM, Amtmann D (2015): The relationship of age, function, and psychological distress in multiple sclerosis. *Psychology, health & medicine*. 20:629-634.

10. Colasanti A, Guo Q, Muhlert N, Giannetti P, Onega M, Newbould RD, *et al.* (2014): In Vivo Assessment of Brain White Matter Inflammation in Multiple Sclerosis with 18F-PBR111 PET. *J Nucl Med*.

11. Politis M, Giannetti P, Su P, Turkheimer F, Keihaninejad S, Wu K, *et al.* (2012): Increased PK11195 PET binding in the cortex of patients with MS correlates with disability. *Neurology*. 79:523-530.

12. Chwastiak L, Ehde DM, Gibbons LE, Sullivan M, Bowen JD, Kraft GH (2002): Depressive symptoms and severity of illness in multiple sclerosis: epidemiologic study of a large community sample. *The American journal of psychiatry*. 159:1862-1868.

13. Smith SM, Zhang Y, Jenkinson M, Chen J, Matthews PM, Federico A, *et al.* (2002): Accurate, robust, and automated longitudinal and cross-sectional brain change analysis. *NeuroImage*. 17:479-489.

14. Patenaude B, Smith SM, Kennedy DN, Jenkinson M (2011): A Bayesian model of shape and appearance for subcortical brain segmentation. *NeuroImage*. 56:907-922.

15. Smith SM, Jenkinson M, Woolrich MW, Beckmann CF, Behrens TE, Johansen-Berg H, *et al.* (2004): Advances in functional and structural MR image analysis and implementation as FSL. *NeuroImage*. 23 Suppl 1:S208-219.
